# Can bioimpedance cardiography assess hemodynamic response to passive leg raising in critically ill patients: A STROBE-compliant study

**DOI:** 10.1097/MD.0000000000023764

**Published:** 2020-12-18

**Authors:** Li Li, Yuhang Ai, Li Huang, Meilin Ai, Qianyi Peng, Lina Zhang

**Affiliations:** Department of Critical Care Medicine, Xiangya Hospital, Central South University, Changsha, China.

**Keywords:** bioimpedance cardiography, cardiac output monitoring, fluid responsiveness, passive leg raising

## Abstract

Passive leg raising (PLR) is a convenient and reliable test to predict fluid responsiveness. The ability of thoracic electrical bioimpedance cardiography (TEB) to monitor changes of cardiac output (CO) during PLR is unknown.

In the present study, we measured CO in 61 patients with shock or dyspnea by TEB and transthoracic echocardiography (TTE) during PLR procedure. Positive PLR responsiveness was defined as the velocity-time integral (VTI) ≥10% after PLR. TTE measured VTI in the left ventricular output tract. The predictive value of TEB parameters in PLR responders was tested. Furthermore, the agreement of absolute CO values between TEB and TTE measurements was assessed.

Among the 61 patients, there were 28 PLR-responders and 33 non-responders. Twenty-seven patients were diagnosed with shock and 34 patients with dyspnea, with 55.6% (15/27) and 54.6% (18/34) non-responders, respectively. A change in TEB measured CO (ΔCO) ≥9.8% predicted PLR responders with 75.0% sensitivity and 78.8% specificity, the area under the receiver operating characteristic curve (AUROC) was 0.79. The Δd^2^*Z*/d*t*^2^ (a secondary derivative of the impedance wave) showed the best predictive value with AUROC of 0.90, the optimal cut point was −7.1% with 85.7% sensitivity and 87.9% specificity. Bias between TEB and TTE measured CO was 0.12 L/min, and the percentage error was 65.8%.

TEB parameters had promising performance in predicting PLR responders, and the Δd^2^*Z*/d*t*^2^ had the best predictive value. The CO values measured by TEB were not interchangeable with TTE in critically ill settings.

## 1 Introduction

Fluid intake and removal are common practice procedures in the intensive care unit (ICU) and associated with increased morbidity and mortality. Fluid intake ensures the delicate volume equilibrium necessitates and reproducible monitoring of volume status and fluid responsiveness. Passive leg raising (PLR) test is the most commonly used method to identify fluid responsiveness with high reliability in various clinical settings.^[1–3]^ Nonetheless, the profound diagnostic performance of PLR requires real-time and accurate monitoring of cardiac output (CO). The classic available tools, including a pulmonary arterial catheter, transpulmonary thermodilution, and echocardiographic Doppler are either invasive or operator-dependent and requiring a learning curve. Therefore, the PLR is still not used globally.^[1,4]^ In theory, the ideal CO monitoring in humans should be non- or mini-invasive, continuous, automated, operator-independent, and so on.^[5]^ None of the currently available devices fulfills those characteristics, while thoracic electrical bioimpedance cardiography (TEB) or bioreactance has the potential to meet the standard criteria.^[5]^

Numerous studies have demonstrated conflicting results about the accuracy of TEB or bioreactance measurements, especially for the absolute values of CO in comparison with thermodilution or echocardiography. In clinical practice, it is more crucial to accurately detect CO changes following the treatment, such as fluid resuscitation, which is the cornerstone of hemodynamic management. Unlike bioreactance, which measures phase shift, TEB measures the amplitude of an oscillating current. Bioreactance's reliability to detect PLR responders has been tested to be acceptable. Nevertheless, whether TEB can accurately follow rapid changes of CO is still unknown. If it is possible, TEB may be compelling to monitor the real-time CO changes. To the best of our knowledge, the utility of TEB is not investigated previously to predict the fluid responsiveness through PLR. The primary objective of the present study was to test the trending ability of the TEB parameters in predicting PLR responders. The secondary objective was to assess the agreement of absolute CO values between TEB and echocardiographic measurements.

## 2 Materials and methods

### 2.1 Patients

The current single-center prospective and observational study was conducted in a 33-bed medical ICU of Xiangya Hospital from June 1 to November 30, 2019. The study was approved by our local institutional review board (Ethics Committee of Xiangya Hospital, Central South University). Written informed consent was obtained from the study participants or their legal representatives.

The inclusion criteria were: age ≥18 years, indications for fluid resuscitation or removal as decided by the attending physicians based on at least one of the following signs:

1. shock, mainly manifested as hypotension (systolic arterial pressure <90 mm Hg or mean arterial pressure (MAP) <65 mm Hg) and/or the presence of inadequate tissue perfusion, that is, mottled skin, oliguria, metabolic acidosis (pH < 7.35 and base excess < −5 mmol/L) or elevated lactate (>2 mmol/L).
2. dyspnea, mainly manifested as a respiratory failure (defined as PaO_2_/FiO_2_ ratio <300) requiring oxygen therapy, including oxygen mask, high flow cannula, invasive, or non-invasive mechanical ventilation.

Patients were excluded if there was active bleeding causing hemodynamic instability, orthopnea unable to lie down, moderate, to massive pleural effusion, systemic edema, intra-abdominal hypertension (bladder pressure >12 mm Hg), maternal situation, pelvic, and/or lower extremity trauma/amputation preventing PLR, unavailable trans-thoracic echocardiography views, severe valvular heart disease, moderate tachyarrhythmia (ventricular rate >120 beats/min) and intracranial hypertension (>20 mm Hg) during the data collection period.

### 2.2 TEB measurements

The TEB system (CSM3100, Cheers Sails Medical, Shenzhen, China) requires the placement of four double electrode sensors on the skin. Upper sensors were placed on the base of the bilateral neck (lateral margin of sternocleidomastoid muscle) and lower sensors at the costal margin of the thorax (mid-axillary line). The outer electrodes in each electrode pair delivered an alternating current of low intensity and high-frequency collected by the inner electrode pair. Changes in thoracic pulsatile blood volume altered the magnitude modulation between currents and were continuously recorded as impedance curves.^[5]^ A proprietary algorithm computes CO from the changes in magnitude. The average value of CO during the past 30 s was displayed on the device screen. Other directly derived parameters, including thoracic fluid content (TFC), left ventricular ejecting time (LVET), d*Z*/d*t* (the slope of the impedance wave) and d^2^*Z*/d*t*^2^ (a secondary derivative of d*Z*/d*t*) were collected to analyze the predictive value. LVET was corrected using Wodey's formula by heart rate (HR): LVET_corrected_ = LVET_measured_ + 1.29 × (HR-60).^[6]^

### 2.3 Focused echocardiography

During the PLR procedure, transthoracic echocardiography (TTE) was performed by an experienced intensivist certified in advanced point-of-care echocardiography. The intensivist was blinded to the TEB results. The quantitative measurements corresponded to comprehensive TTE guidelines.^[7]^ From the parasternal long-axis view, the left ventricular outflow tract (LVOT) diameter was measured at 5 mm behind the aortic valve in mid-systole, which was used to auto calculate the LVOT area. From the apical five-chamber plane, LVOT velocity-time integral (VTI) was measured by Doppler tracing at the end of expiration. To ensure precise measurement of VTI, each measurement was performed in triplicate and averaged in sinus rhythm, while five measurements were averaged in arrhythmia.^[8,9]^ CO was calculated as averaged VTI multiplied by LVOT area and HR at baseline and 1 min after PLR.

### 2.4 Study procedure and data collection

To ensure minimal variability, the PLR and data collection were performed only by the technicians, who were provided with in-depth training on the TEB technology by the manufacturer. The patients who received mechanical ventilation were given analgesic and sedation drugs to maintain a Critical-care pain observation tool (CPOT) score of 0 to 2 and a Richmond agitation-sedation scale (RASS) score of −2 to 0. For the spontaneous breathing patients, the practitioner explained the procedure and acquired informed consent. Subject demographic characteristics were recorded, including Simplified Acute Physiological Score II, Sequential Organ Failure Assessment, medical history, oxygenation index, lactate level, admission diagnosis category, the presence of vasopressor/inotrope, and mechanical ventilation.

The following vital signs were monitored and recorded during the procedure: HR, MAP, and pulse pressure (PP). At first, the patient was positioned at 45° semi-recumbent position. After achieving a stable hemodynamic signal on the TEB system, the baseline hemodynamic data, including VTI were collected. Then, the remote control of the bed was used to tilt the trunk to a horizontal position, and the legs were tilted 45° upwards.^[2]^ The TEB recording and VTI measurements were performed at the same time, both of which were ∼60 to 90 s after PLR. The time lag was previously validated, corresponding to the maximum change in CO following the PLR maneuver.^[10]^ The changes of hemodynamic parameters in two positions were presented as percentages, for example, ΔHR = (HR after PLR − HR at baseline) / HR at baseline × 100%, ΔVTI = (VTI after PLR − VTI at baseline) / VTI at baseline × 100%. PLR responder was defined as ΔVTI ≥ 10%.^[11]^ If ΔHR was >10%, the procedure was regarded as inadequate, and the patient's data was excluded.

### 2.5 Statistical analysis

All statistical analyses were performed with MedCalc 17.6 (MedCalc Software, Mariakerke, Belgium) and SPSS 22.0 (IBM, Armonk, NY). Assuming that the area under the receiver operating characteristic curve (AUROC) was at least 0.80, α = 0.01 and β = 0.05, 28 patients in each group were required. The Kolmogorov–Smirnov test was used to analyze the normality of continuous data. The data were expressed as mean (standard deviation, SD), median (interquartile range), or number (frequency in %). A comparison between PLR responders and non-responders was assessed using the two-sample Student's *t* test or the Mann–Whitney *U* test, depending on the data distribution. The AUROC was used to determine the sensitivity and specificity of TEB parameters to predict the PLR responsiveness. To assess the concordance of absolute CO values between TEB and TTE measurements, the Pearson correlation coefficient was used with a 95% confidence interval (CI). Their agreement was performed by using Bland-Altman analysis. The percentage error was calculated as 2SD divided by the mean of TEB, and TTE measured CO values.^[12]^*P* < .05 was considered as statistical significance.

## 3 Results

### 3.1 Patient characteristics

**Figure 1:**
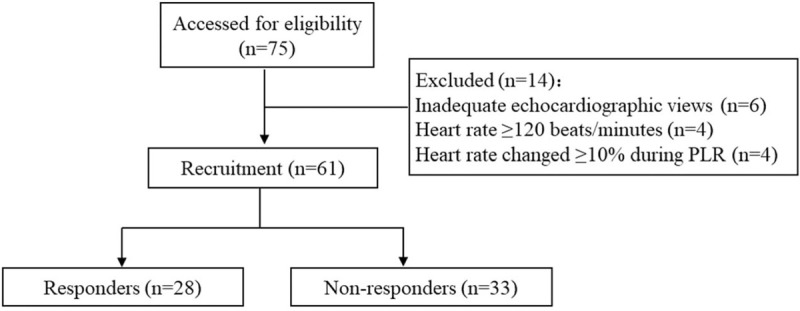
Flowchart of patient recruitment. PLR = passive leg raising.

A total of 75 patients were screened for inclusion. Among them, 14 patients were excluded because 6 patients did not have appropriate TTE views, 4 patients had HR more than 120 beats/min, and 4 patients had ΔHR >10% during PLR procedure (Fig. 1). Among the included 61 patients, 28 patients were PLR responders, and 33 patients were non-responders. There were no statistical differences in the demographic characteristics and illness severity between PLR responders and non-responders (Table 1). Twenty-seven patients were diagnosed with shock and 34 patients with dyspnea, with 55.6% (15/27) and 52.9% (18/34) non-responders. The majority of patients in the overall population were on assisted mechanical ventilation. The non-responders tended to lower the oxygenation index than the PLR responders (249.39 ± 151.07 vs 319.29 ± 137.90, *P* = .07), though not statistically significant. This trend suggests that these patients were intravascularly volume overloaded, resulting in their lower oxygenation index and attenuated responsiveness to PLR.

**Table 1:** The characteristics of baseline patients.

Parameters	Overall patients	PLR responders	PLR non-responders	*P*
Number, n	61	28	33	
Men, n (%)	34 (55.7)	18 (64.3)	16 (48.5)	.30
Age, year	60.31 ± 15.55	59.86 ± 14.45	60.70 ± 15.87	.84
BMI (kg/m^2^)	1.62 ± 0.16	1.63 ± 0.17	1.61 ± 0.16	.55
APACHE II score	26.16 ± 10.82	27.04 ± 10.64	25.42 ± 11.07	.57
SOFA score	9.56 ± 5.20	9.75 ± 5.62	9.39 ± 4.89	.79
Medical history, n				
Coronary heart disease	14	7	7	.73
Hypertension	23	10	13	.80
Diabetes	14	5	9	.38
Others	17	7	10	.65
None of the above	25	13	12	.43
Etiology				.84
Shock, n (%)	27 (44.3)	12 (42.9)	15 (45.5)	
Acute dyspnea, n (%)	34 (55.7)	16 (57.1)	18 (54.5)	
Mechanical ventilation, n (%)	45 (73.8)	21 (75.0)	24 (72.7)	.84
PaO_2_/FiO_2_	281.47 ± 148.19	319.29 ± 137.90	249.39 ± 151.07	.07
Lactate (mmol/L)	1.60 (1.00, 3.10)	2.20 (1.10, 3.45)	1.40 (0.83, 2.80)	.20
Norepinephrine, n (%)	25 (41.0)	11 (39.3)	14 (42.4)	.80

### 3.2 Predictive values of TEB parameters

The AUROC of TEB measured ΔCO for determining PLR responders was 0.79 (95% CI: 0.67–0.91, *P* < .001), the optimal cut point was ≥9.8% with 75.0% sensitivity and 78.8% specificity. Among the key TEB parameters, Δd^2^*Z*/d*t*^2^ showed the best predictive value with AUROC of 0.90 (95%CI: 0.82–0.98, *P* < .001), the optimal cut point was ≥ −7.1% with 85.7% sensitivity and 87.9% specificity. Other TEB parameters showed varying predictive performance of the PLR test, as reflected by AUROC ranging from 0.47 to 0.79 (Table 2). The changes in MAP and PP during PLR had poor predictive value with AUROC of 0.55 (95% CI: 0.41–0.70, *P* = .48) and 0.62 (95% CI: 0.48–0.77, *P* = .1), respectively.

**Table 2:** Predictable value of changes of TEB parameters and vital signs.

Parameters	AUROC	95% confidence interval	*P*	Optimal cutoff (%)	Sensitivity (%)	Specificity (%)	PPV (%)	NPV (%)
ΔCO_TEB_	0.79	0.67–0.91	.00^∗^	9.8	75.0	78.8	75.0	78.8
ΔTFC	0.47	0.33–0.62	.70	6.7	27.3	89.3	25.0	49.0
Δd*Z*/d*t*	0.90	0.82–0.98	.00^∗^	−3.6	85.7	87.9	85.7	87.9
Δd^2^*Z*/d*t*^2^	0.90	0.82–0.98	.00^∗^	−7.1	85.7	87.9	85.2	85.3
ΔLVETcorr	0.79	0.66–0.91	.00^∗^	1.7	85.7	75.8	75.0	86.2
ΔMAP	0.55	0.41–0.70	.48	−3.5	85.7	39.4	50.0	64.7
ΔPP	0.62	0.48–0.77	.10	−0.8	71.4	54.5	45.7	53.8

### 3.3 Relative changes of hemodynamic measurements during PLR procedure

Based on TEB measurements, the mean CO changes in the PLR responders were significantly greater than non-responders (10.4 ± 16.0% vs −4.9 ± 9.5%, *P* < .001) (Table 3). The Δd^2^*Z*/d*t*^2^ and ΔLVET_corr_ increased significantly in PLR responders (13.0 ± 14.5 vs −14.0 ± 13.5 and 3.7 ± 4.6% vs −0.4 ± 4.0%, respectively, *P* < .001). The PLR responders had a tendency of lower baseline d*Z*/d*t* (4.2 ± 0.9 vs 4.7 ± 1.1, *P* = .05) and d^2^*Z*/d*t*^2^ (18.3 ± 7.9 vs 23.5 ± 11.9, *P* = .05). The baseline and changes of vital signs, including HR, PP, and MAP had no significant difference between PLR responders and non-responders.

**Table 3:** Hemodynamic measurements during PLR procedure.

Parameters	Fluid responsiveness (n = 28)	No fluid responsiveness (n = 33)	*P*
Heart rate (HR, beats/min)			
Baseline	92.89 ± 19.33	90.15 ± 17.62	.57
After PLR	91.18 ± 19.09	89.58 ± 17.47	.73
ΔHR during PLR (%)	0.97 ± 3.39	1.28 ± 4.08	.75
Mean arterial pressure (MAP, mm Hg)			
Baseline	82.79 ± 17.53	87.48 ± 17.59	.30
After PLR	84.39 ± 14.20	87.68 ± 17.73	.43
ΔDBP during PLR (%)	2.71 ± 11.44	1.48 ± 12.08	.69
Pulse pressure (PP, mm Hg)			
Baseline	58.93 ± 27.60	55.91 ± 22.07	.64
After PLR	58.25 ± 24.85	55.73 ± 21.09	.67
ΔPP during PLR (%)	9.25 ± 26.87	−2.96 ± 19.92	.05
CO_TTE_ (transthoracic echocardiographic cardiac output, L/min)			
Baseline	3.73 ± 2.00	4.17 ± 1.79	.38
After PLR	4.15 ± 2.15	4.35 ± 1.86	.71
ΔCO_TTE_ during PLR (%)	16.92 ± 5.78	1.09 ± 7.19	.00^∗^
CO_TEB_ (thoracic electrical bioimpedance cardiography measured cardiac output, L/min)			
Baseline	4.10 ± 1.38	4.32 ± 1.43	.54
After PLR	4.42 ± 1.32	4.11 ± 1.39	.38
ΔCO_TEB_ during PLR (%)	10.41 ± 15.99	−4.91 ± 9.47	.00^∗^
d*Z*/d*t* (slope of the impedance wave, Ω/s)			
Baseline	4.19 ± 0.87	4.72 ± 1.12	.05
After PLR	4.42 ± 0.80	4.36 ± 1.03	.80
Δd*Z*/d*t* during PLR (%)	6.10 ± 6.89	−7.52 ± 7.07	.00^∗^
d^2^*Z*/d*t*^2^ (secondary derivate of d*Z*/d*t*, Ω/s^2^)			
Baseline	18.29 ± 7.86	23.48 ± 11.94	.05
After PLR	20.13 ± 7.42	20.03 ± 9.72	.96
Δd^2^*Z*/d*t*^2^ during PLR (%)	13.02 ± 14.48	−13.98 ± 13.53	.00^∗^
LVET_corr_ (corrected left ventricular ejection time, ms)			
Baseline	289.53 ± 39.03	279.57 ± 48.36	.39
After PLR	299.60 ± 38.80	278.05 ± 45.94	.06
ΔLVET_corr_ during PLR (%)	3.65 ± 4.61	−0.36 ± 3.97	.001

### 3.4 The concordance between absolute TEB and TTE measured CO values

**Figure 2:**
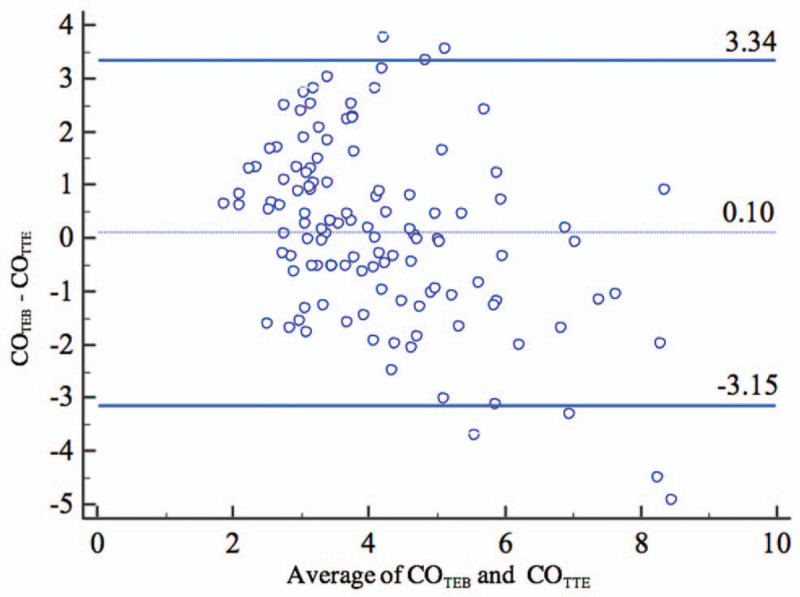
Bland–Altman plots for comparisons of absolute values of cardiac output (n = 122 pairs). CO_TEB_ = cardiac output measured by thoracic electrical bioimpedance cardiography, CO_TTE_ = cardiac output measured by transthoracic echocardiography.

One hundred twenty-two paired comparisons were performed between TEB and TTE measured CO values. Absolute CO values were significantly correlated (*r* = 0.71, n = 122, *P* < .001). In the Bland-Altman plot analysis, the mean difference between TEB and TTE measured CO values in the overall population was 0.12 L/min (95% limits of agreement: −2.56 to 2.81 L/min), and the percentage error was 65.8% (Fig. 2). The CO measurements at baseline (n = 61 paired comparisons) showed a bias of 0.18 L/min between TEB and TTE with 67.6% percentage error; however, after PLR (n = 61 paired comparisons) the bias was 0.05 L/min with 64.0% percentage error.

## 4 Discussion

This study reported that noninvasive TEB had significant predictive value in determining PLR responders in critically ill patients, although the absolute CO values were not interchangeable between TEB and TTE measurements.

TEB has theoretical advantages and wide clinical applications for CO monitoring due to its advantage of noninvasiveness, convenience, and simplicity. Numerous studies have focused on TEB precision for absolute CO values, but conflicting results were reported. The correlation coefficient of TEB with bolus thermodilution was significant, with overall *r* ranging from 0.79 to 0.82.^[5]^ However, the agreement between the two techniques was not good. In a consecutive meta-analysis, compared with thermodilution methods, the percentage error for TEB ranged from 37% to 42% in perioperative patients.^[4,8,13]^ The percentages were above a clinically acceptable threshold of 30% when comparing agreements between two CO measurement techniques.^[4]^ The present study showed more than 60% percentage of TEB error compared with TTE measurements. The poor concordance might be attributed to the different reference methods and invalidity of TEB devices. There are several factors limiting the validity of TEB measurements.^[5,14]^ One is the physiological and pathophysiological situations, including pregnancy, obesity, and pleural effusion. Another is the patients’ spontaneous movements such as heart rhythm, respiratory effort, and mechanical ventilation. Furthermore, the highly equipped ICU setting was demonstrated to increase the level of noise. All of the factors may interfere with the low signal/noise ratio of TEB, resulting in artifacts and invalid measurements. In fact, the precision of TEB measured CO had been reported to be poorer in ICU settings than in traditional medicine department.^[15]^

Considering that the interfering factors affecting the validity of TEB measurements were almost fixed during the PLR procedure, we postulated that TEB could track the CO changes and predict PLR responders with good discrimination. TEB was previously demonstrated to be an unreliable method of tracking CO changes during hemodynamic load challenge in healthy volunteers.^[16]^ Comparing absolute values and percent changes of CO across three timepoints, the researchers found that TEB measured CO had a weak correlation and poor agreement with TTE.^[16]^ However, the disagreement of TEB measured CO with reference method did not exclude the ability to predict PLR responders. A novel bioimpedance device, endotracheal bioimpedance cardiography (ECOM) has improved signal/noise ratio with bioimpedance electrodes close to the ascending aorta. ECOM has shown promising predictive value with AUROC of 0.81 in determining PLR responders,^[17]^ even though the CO values measured by ECOM were not interchangeable with pulse contour analysis, and the percentage error was 45%.^[16]^ Another device, known as non-invasive CO monitor (NICOM) has also shown poor agreement with thermodilution^[18,19]^ but good consistency in determining PLR responders^[20]^ in critically ill patients. Factually meta-analysis of PLR tests showed no difference in diagnostic performance among various measurement techniques, including pulse contour analysis, bioreactance and Doppler (both transesophageal and transthoracic methods).^[3]^ Despite the poor agreements, the CO monitoring device, which is able to identify fluid responder is clinically valuable due to its noninvasiveness and convenience.

In addition, our study showed that several key TEB parameters, which were directly measured by TEB, had varying performances in predicting PLR responders. Among these, d^2^*Z*/d*t*^2^, which represented the acceleration rather than the amount of aortic blood flow, had the greatest AUROC value. d^2^*Z*/d*t*^2^ was introduced as a new algorithm for noninvasive determination of CO,^[21]^ which is called electrical velocimetry (EV). Though the previous study yielded diverging results about the accuracy of EV in CO measurements^[22]^; however, a recent study demonstrated it was reproducible under different loading conditions.^[23]^ The present study was the first one to describe the trends of d^2^*Z*/d*t*^2^ and its predictive value in determining PLR responders. Recent studies have documented that the change of LVET_corrected_ and the time required for ejection in the cardiac cycle was well correlated with the changes in volume status during PLR, fluid challenge, or dialysis.^[24–26]^ Mechanical ventilation, respiratory rate, and high positive end-expiratory pressure had no significant impact on the predictive value of LVET_corrected_. In previous studies,^[24–26]^ LVET was measured by carotid Doppler in the form of carotid flow time. The present study revealed that TEB measured LVET can be translated into clinical application due to its convenience. In contrast, TFC, which is inversely proportional to base impedance (*Z*_0_), was worthless in predicting PLR responders. *Z*_0_ depends on the amount of intrathoracic fluid volume, the distance between electrodes, and so on. The invalidity and insignificant change of TFC during PLR may be the attributed factors to its worthlessness. Above all, the different performances of LVET, d^2^*Z*/d*t*^2^ and TFC may explain the relatively low predictive value of TEB measured CO in identifying PLR responders. Since the TEB-measured CO was calculated from the multiplicative model composed of LVET, d*Z*/d*t*, TFC, and a calibration factor. The present study may provide clues to improve the technology and algorithms of TEB in the future.

This study had the following limitations. The fluid challenge was not used as a reference standard to identify fluid responsiveness to avoid potentially inefficient yet detrimental volume overload in some of our patients. Therefore, the trending ability of TEB in tracking CO changes cannot be further determined during fluid resuscitation or administration of vasoactive medication. We used echocardiographic CO as the reference rather than thermodilution methods to conveniently collect the samples. VTI change was selectively chosen to define PLR responders,^[11]^ because it was simpler than CO measurement and avoided the intra-observer variation of LVOT measurement. To reduce the high variability of Doppler-based measurements, VTI tracing was averaged three or five times, respectively, for sinus rhythm and arrhythmia, which has been proven to be enough to obtain an acceptable precision (interquartile range highest value <10%) for VTI.^[9]^ Therefore, the high percentage error of TEB in the present study was statistically significant but unacceptable. This study may provide clues for the improvement of technology and algorithms to reach a sufficient reproducibility of TEB.

In summary, the TEB device, although not interchangeable with TTE for CO measurements, can predict PLR responders with good discrimination in critical settings. Among the key TEB parameters, d^2^*Z*/d*t*^2^ had the highest predictive value in determining PLR responders.

## Acknowledgments

We thank the National Clinical Research Center for Geriatric Disorders (Xiangya Hospital).

## Author contributions

**Conceptualization:** Qianyi Peng, Lina Zhang.

**Data curation:** Li Li, Qianyi Peng.

**Formal analysis:** Yuhang Ai.

**Funding acquisition:** Lina Zhang.

**Investigation:** Yuhang Ai.

**Methodology:** Yuhang Ai, Meilin Ai.

**Project administration:** Li Li, Qianyi Peng, Lina Zhang.

**Resources:** Li Huang, Lina Zhang.

**Software:** Li Huang, Meilin Ai.

**Supervision:** Li Huang.

**Validation:** Qianyi Peng.

**Visualization:** Meilin Ai.

**Writing – original draft:** Li Li.

**Writing – review & editing:** Lina Zhang.
